# Identification and Clonal Characterisation of a Progenitor Cell Sub-Population in Normal Human Articular Cartilage

**DOI:** 10.1371/journal.pone.0013246

**Published:** 2010-10-14

**Authors:** Rebecca Williams, Ilyas M. Khan, Kirsty Richardson, Larissa Nelson, Helen E. McCarthy, Talal Analbelsi, Sim K. Singhrao, Gary P. Dowthwaite, Rhiannon E. Jones, Duncan M. Baird, Holly Lewis, Selwyn Roberts, Hannah M. Shaw, Jayesh Dudhia, John Fairclough, Timothy Briggs, Charles W. Archer

**Affiliations:** 1 Cardiff School of Biosciences, Cardiff University, Cardiff, United Kingdom; 2 Department of Pathology, Cardiff University, Cardiff, United Kingdom; 3 Cytogenetics Department, University Hospital of Wales, Cardiff, United Kingdom; 4 Department of Veterinary Clinical Sciences, The Royal Veterinary College, North Mymms, United Kingdom; 5 Department of Orthopaedics, University Hospital of Wales, Cardiff, United Kingdom; 6 Royal National Orthopaedic Hospital, Stanmore, United Kingdom; Ohio State University, United States of America

## Abstract

**Background:**

Articular cartilage displays a poor repair capacity. The aim of cell-based therapies for cartilage defects is to repair damaged joint surfaces with a functional replacement tissue. Currently, chondrocytes removed from a healthy region of the cartilage are used but they are unable to retain their phenotype in expanded culture. The resulting repair tissue is fibrocartilaginous rather than hyaline, potentially compromising long-term repair. Mesenchymal stem cells, particularly bone marrow stromal cells (BMSC), are of interest for cartilage repair due to their inherent replicative potential. However, chondrocyte differentiated BMSCs display an endochondral phenotype, that is, can terminally differentiate and form a calcified matrix, leading to failure in long-term defect repair. Here, we investigate the isolation and characterisation of a human cartilage progenitor population that is resident within permanent adult articular cartilage.

**Methods and Findings:**

Human articular cartilage samples were digested and clonal populations isolated using a differential adhesion assay to fibronectin. Clonal cell lines were expanded in growth media to high population doublings and karyotype analysis performed. We present data to show that this cell population demonstrates a restricted differential potential during chondrogenic induction in a 3D pellet culture system. Furthermore, evidence of high telomerase activity and maintenance of telomere length, characteristic of a mesenchymal stem cell population, were observed in this clonal cell population. Lastly, as proof of principle, we carried out a pilot repair study in a goat *in vivo* model demonstrating the ability of goat cartilage progenitors to form a cartilage-like repair tissue in a chondral defect.

**Conclusions:**

In conclusion, we propose that we have identified and characterised a novel cartilage progenitor population resident in human articular cartilage which will greatly benefit future cell-based cartilage repair therapies due to its ability to maintain chondrogenicity upon extensive expansion unlike full-depth chondrocytes that lose this ability at only seven population doublings.

## Introduction

Articular cartilage displays a poor repair capacity. Consequently, the aim of cartilage cell therapy procedures is to repair damaged joint surfaces with a functional replacement tissue. As an avascular tissue, cartilage comprises a single cell type – the chondrocyte, which is organised into three distinct layers – the surface, mid and deep zones [1]. Chondrocytes required for cell-based therapies are isolated and expanded *in vitro* to generate sufficient numbers of cells for surgical procedures. However, extensive expansion results in the progressive dedifferentiation of the chondrocytes. In culture, human chondrocytes show an inability to retain a chondrogenic potential past 7 population doublings, even after cultivation in a chondrogenically permissive environment [2], [3], [4]. In order to combat this problem of dedifferentiation, research has focussed on the use of growth factors and 3D culture systems as a way of maintaining the chondrogenic potential of these cells [5], [6], [7], [8]. Although these modifications, to some extent, have proved successful they would be unsuitable as a method of expanding cells for use in cell-based repair therapies and, as such, monolayer culture is a limiting factor for chondrocyte efficacy. Additionally, when chondrocytes are used in cell-based tissue engineering, the resulting repair tissue is unpredictable and often fibrocartilagenous. It is argued that this fibrocartilage is biochemically and biomechanically inferior to native cartilage thus compromising long-term repair of the cartilage defect [9], [10], [11].

The loss of the chondrogenic phenotype during monolayer culture means that the size of defect that can be treated is limited since only a defined amount of cartilage can be harvested from the joint periphery. One way to overcome this cell source limitation would be to use an alternative cell type that maintains its inherent proliferative capacity, such as a mesenchymal stem cell (MSC) population [12]. Recently, studies have demonstrated that cells obtained from a range of adult tissues eg. adipose, epidermal, dental pulp and bone marrow exhibit mesenchymal/progenitor type properties; they can differentiate into multiple lineages and express putative stem cell markers and as such, could be used for cell-based repair therapies [13], [14], [15], [16], [17], [18], [19], [20]. In particular, studies have highlighted that MSCs obtained from bone marrow could be used in cartilage repair procedures as bone marrow stromal cells (BMSC) can be directed towards the chondrogenic lineage [21]. However, articular cartilage is a permanent cartilage and the phenotype generated by BMSCs is endochondral, which will terminally differentiate, an unfavourable outcome if one wants to repair permanent articular cartilage. At present, the type of cartilage generated by stem cells from other tissue types is poorly characterised [22], [23].

Instead of utilising MSCs from different tissue sources for cartilage repair strategies, it would be logical to use cells from the same tissue as these cells may possess the developmental repertoire of the native tissue, impacting on the morphogenesis of the repair tissue in a beneficial way. Previously, research has hypothesised that foetal and immature articular cartilage develops appositionally – that is from the surface to the deep zone [24]. In support of these data, the identification of a chondroprogenitor population in the surface of bovine articular cartilage has been described and this has opened up the possibility that human cartilage could also contain a cartilage progenitor population [25], [26]. Identification and characterisation of this cartilage progenitor population in human tissue has begun in earnest. Many studies relying on the use of several putative stem cell markers to determine a method by which these cells can be isolated from a full-depth articular chondrocyte population [27], [28], [29]. As of yet, however, no suitable single marker has been established and it is becoming increasingly evident that a high percentage of mature chondrocytes also express these markers thus confounding previous studies, especially in the absence of clonal analysis [30], [31]. As cell surface marker expression can alter in monolayer culture, then it is important that other classical stem cell characteristics are relied on to determine whether a true cartilage progenitor population has been isolated [31], [32].

This study describes the isolation of a distinct cartilage progenitor population resident in normal human cartilage of varying ages. The methodologies employed result in the generation of large numbers of cells from a single cell. Upon characterisation of this cell population derived from a single cell, we demonstrate its progenitor-like properties. As such, we propose that the human cartilage progenitor population characterised in this study is a suitable candidate for advancing cell-based tissue repair therapies for cartilage defects.

## Materials and Methods

### Ethics Statement

Cartilage tissue samples were obtained from patients who underwent knee surgery. South East Wales NHS Research Ethics Committee specifically approved this study and institutional safety and ethical guidelines were followed. Written informed consent was obtained from each patient, and extensive precautions were taken to preserve the privacy of the participants donating tissue. All caprine *in vivo* studies were approved by the Office for Food Safety and Animal Health Graubunden, permitting the project to take place under the title PROCART. Experiments were carried out under the strict guidelines of the AO Research Institute, Switzerland.

### Cell isolation & fibronectin adhesion assay

Full-depth normal human articular cartilage samples from femoral chondyles (n = 9; mean age 30.0 yrs, range 10–57) were obtained. Chondrocytes were isolated by sequential pronase (70 U ml^−1^, 1 hour at 37°C) and collagenase (300 U ml^−1^, 3 hours at 37°C) digest. Isolated cells were plated down as a full-depth chondrocyte population (total cell mass from surface, mid and deep zones) or subjected to a fibronectin adhesion assay as described [33]. Briefly, six well plates were coated with 10 µg ml^−1^ fibronectin (FN; Sigma, UK) in 0.1 M phosphate buffered saline (PBS, pH7.4) containing 1 mM MgCl and 1 mM CaCl_2_ (PBS+) overnight at 4°C. Control dishes were treated with PBS+ containing 0.1% BSA overnight at 4°C. Isolated full-depth chondrocytes (4000 cells ml^−1^) were seeded onto the coated plates for 20 mins at 37°C in Dulbeccos Modified Eagle Medium (DMEM). In all experiments, a minimum of 3 fibronectin and 3 BSA coated dishes were used. After 20 mins, media and non-adherent cells were removed. Fresh DMEM containing Penicillin 10000 µg ml^−1^/Streptomycin 10000 U ml^−1^, 0.1 mM ascorbic acid, 0.5 mg ml^−1^ L-glucose, 100 mM HEPES, 1 mM sodium pyruvate, 2 mM L-glutamine and 10% fetal bovine serum (FBS) (DMEM+) were added to the remaining adherent cells. Within 18 hours of plating, the number of cells adhered was counted.

Twelve days after plating, colonies (defined as a cluster of more than 32 cells, as this represents a population of cells derived from more than 5 population doublings of a single cell, thereby discounting a transit amplifying cell cohort) were counted and colony forming efficiency calculated [33]. Colonies were selected and isolated using sterile cloning rings (Sigma, UK). Colonies were expanded in DMEM+ plus 1 ng ml^−1^ Transforming Growth Factor – β2 (TGF-β2; PeproTech, UK) and 5 ng ml^−1^ Fibroblast Growth Factor-2 (FGF-2; PeproTech, UK). At each passage, the number of cells obtained and re-plated was recorded. From these data, population doublings from 4 specimens, with at least 3 clones from each specimen, were calculated using the equation: n  =  [log (final cell count) - log (number of cells initially plated)]/0.301 [34].α

### Flow cytometry

One million full-depth chondrocytes (passage 5) were washed in PBS and incubated for 1 hour at 4°C with conjugated antibodies to CD105-FITC (Ancell), CD166-RPE (Ancell), CD44-FITC (Pharminogen), CD29-FITC (Chemicon) or CD49e-PE (Chemicon) at a concentration of 10 µg ml^−1^. Cells were centrifuged at 2000×g, supernatants removed and cells washed three times in PBS. Finally, labelled cells were re-suspended in 1 ml PBS and subjected to single channel Fluorescently Activated Cell Sorting (FACS) analysis. The appropriate IgG controls were run in parallel. A minimum of three clones were analysed for each antibody.

### Immunocytochemistry

Monolayer cultures of full-depth chondrocytes and cartilage progenitors were fixed and subjected to immunofluorescence for antibodies to anti-human Notch 1 (Developmental Studies Hybridoma Bank, USA), anti-human CD90 (BD Pharminogen, USA), anti-human STRO-1 (R&D Systems, USA), anti-rabbit Jagged 1 (Santa Cruz Biotech, USA) and anti-goat Delta 1 (Santa Cruz Biotech). Monolayer cultures subjected to chondrogenic induction were analysed for anti-mouse Sox9 (Abcam, UK), aggrecan-IGD; 6B4+ and 2B6 (both kind gifts of Professor Bruce Caterson, Cardiff University), collagen type I (10 µg ml^−1^; Abcam) and collagen type II (DSHB). Full protocols can be found in supporting information (Text S1). Pellets from a minimum of 3 different clonal cell lines were labelled for each antibody.

### Cell differentiation assays

Expanded clonal cell lines and full-depth chondrocytes were trypsinised and aliquots of 0.5×10^6^ cells per 1 ml of media were centrifuged at 2000×g for 5 mins in 1.5 ml Eppendorf tubes to form spherical pellets. For chondrogenic differentiation, pellets were suspended in DMEM supplemented with ITS (10 µg ml^−1^ insulin, 5.5 µg ml^−1^ transferrin, 5 ng ml^−1^ selenium; GIBCO, UK), 100 mg ml^−1^ Gentamicin, 50 µg ml^−1^ L-ascorbic acid, 1 mg ml^−1^ L-glucose, 2 mM L-glutamine, 10 mM HEPES, 10^−7^ M dexamethasone, 2% FBS and 10 ng ml^−1^ TGF-β2. For osteogenic differentiation pellets were suspended in DMEM, 10% FBS, 10 mM β-glycerophosphate, 10 nM dexamethasone and 0.1 mM L-ascorbic-acid-2-phosphate. Pellets were cultured for 21 days with a medium change every other day.

Monolayer cultures of cartilage progenitor cell lines and full-depth chondrocytes were also established and cultured in chondrogenic differentiation media or adipogenic differentiation was induced using cycles of treatment with adipogenic induction media or adipogenic maintenance media as previously described [35]. Cell monolayers cultured in adipogenic medium were fixed in 10% NBFS for 10 mins. For lipid detection, a stock solution (0.5% Oil Red O in 100% isopropanol) was diluted at a ratio of 4:6 with dH_2_O and the cells incubated for 1 hour.

### Preparation of pellets for analysis

At 21 days, cell pellets were fixed in 4% para-formaldehyde, embedded in paraffin and cross-sectioned at 8 µm or following fixation in 10% neutral buffered formalin, the pellets were fully dehydrated and processed into Technovit 9100 New® (TAAB Laboratories, UK) using the chemical catalytic method fully described by Yang *et al*, [36] with fully destabilised resin at all steps [37]. The pellets embedded in Technovit 9100 New® resin sections were cut using glass knives to a thickness of 1 µm.

### Pellet analysis

Wax embedded pellet sections were dewaxed in xylene and hydrated in a decreasing graded alcohol series. Technovit 9100®New embedded pellets were de-acrylated in 2-methoxyethyl acetate (Sigma, Dortset, UK) for 3–4 hours followed by rehydration through xylene, and a series of graded ethanol. Resin autofluorescence was quenched by immersing tissue sections in 1% sodium borohydride and equilibrated in PBS as previously described [37]. Pellets sections were then subjected to standard histological protocols for safranin O and toluidine blue staining for proteoglycan detection. Alizarin red and von Kossa staining were used to determine the extent of mineralisation and picro-sirius red staining was performed to demonstrate collagen fibril synthesis within the pellet matrix. Immunofluorescent analysis for cartilage (Collagen type I & II, 2B6, aggrecan) and osteogenic (Collagen type X, alkaline phosphatase) components was performed, as described above and more fully in supporting information (Text S1).

### PCR analysis

At 21 days, cell pellets cultured in osteogenic differentiation media were placed into buffer RLT (Qiagen, UK). Cell monolayers from adipogenic cultures were lysed from the tissue plastic using buffer RLT (Qiagen, UK). RNA was extracted from pellet and monolayer cells using a kit (RNAEasy, Qiagen, UK) with a DNAseI incubation step. RNA was quantified using a Nanodrop 2000c spectrophotometer and 100 ng of each sample was used for reverse transcription. Polymerase chain reactions were performed using the following primer combinations for lipo-protein lipase forward 5′CTGAAGACACAGCTGAGGAC3′, reverse 5′CTGGTGAATGTGTGTAAGAC3′, osteonectin forward 5′TCCACAGTACCGGATTCTCTCT3′, reverse 5′TCTATGTTAGCACCTTGTCTCCAG3′, 18S rRNA forward 5′GATGGGCGGCGGAAAATAG3′, reverse 5′GCGTGGATTCTGCATAATGGT3′. PCR reactions were amplified using the following conditions 95°C 2 min-1 cycle, 95°C 30 sec, 53°C 30 sec, 72°C 30 sec-35 cycles, 72°C 5 min-1cycle.

### Cytogenetic analysis

Cytogenetic investigations were undertaken on 1 clonal cell line from 1 specimen (10 year-old female; PD = 31.3) and 2 clonal cell lines from a different specimen (54 year-old female; PD = 23.6 & 28.5). Cultures were selected for harvest when cell growth was almost confluent. Chromosome preparations were obtained using a modification of conventional techniques explained in detail in supporting information (Text S2) [38]. The karyotype was determined by microscopic examination after modification of the Giemsa staining and banding analysis [39], [40].

### Telomere length analysis

Two full-depth chondrocyte populations (1 at PD10 and 1 at PD12) and 3 clonal cell lines (1 at PD 29, the second at PD26 and PD39 and the third at PD29 and PD38), were washed in sterile PBS, pelleted at 1.0×10^6^ cells and frozen in liquid nitrogen for telomere length analysis. DNA extractions and single length telomere analysis (STELA) reactions at the 17p and XpYp telomeres were carried out as described previously and in full in supporting information (Text S3) [41]. Corresponding telomerase activity using real time quantitative repeat amplification procedure (RTQ-TRAP) was carried out for the corresponding full-depth chondrocyte and clonal cell lines, described in full in supporting information (Text S4).

### *In ovo* injections and tissue processing

Cell engraftments: Clonal cells were expanded, harvested, and aliquots of between 1×10^4^–1×10^5^ cells re-suspended in media and immediately injected into the limb bud of 3-day-old (st23) [42] chick embryos which had previously been windowed. Eggs were resealed with adhesive tape and re-incubated up to day 10 (st36–37). Embryos were killed by cervical dislocation, a note of their developmental stage taken and the hind-limbs removed. The limbs were washed in 0.1 M PBS (pH 7.4) and fixed in 10% NBFS for 1 hour at room temperature before being processed to wax. Sections were cut (8 µm) and subjected to immunohistochemistry, protocol previously described, for anti-human collagen-1 (Abcam, UK) at a dilution of 1:1000 to detect engrafted cells. The antibody did not cross-react to chick type I collagens or to other chick collagens. To determine specificity of the antibody, positive controls were carried out on human cartilage tissue sections and negative controls were run on non-injected chick limbs.

*In situ* hybridisation for human-specific Alu genomic repeats: In situ hybridisation for human Alu genomic repeats was performed as described previously [4], [43].

### Caprine *in vivo* repair study

In order to obtain proof-of principle, a pilot study was carried out using 6 young, mature female goats. In the first surgical procedure a circular 6 mm defect was created in the lateral femoral condyle whilst preserving the calcified layer. The protocol followed for cartilage digestion and chondroprogenitor isolation was identical to that described here for human cartilage progenitors. Colony forming efficiency and population doublings for the goat chondroprogenitors were calculated. Cells were pelleted into a 3D culture system and cultured in chondrogenic induction media, as described previously in this manuscript. Immunohistological analysis was performed on goat chondroprogenitor pellets for Collagen type II (10 µg/ml^−1^; DSHB) and aggrecan-IGD;6B4 (king gift from Professor Bruce Caterson, Cardiff University) as described previously. RNA was isolated from the chondrogenic induced pellet cultures, transcribed to cDNA and polymerase chain reaction (PCR) performed to determine Collagen type II and Sox 9 mRNA expression.

Three weeks after the initial isolation, in a second surgical procedure, 2×10^5^ cells were loaded onto a 5 mm diameter type I/III collagen membrane (Chondro-Gide®, *Geistlich AG*). Three goats received a membrane with goat chondroprogenitors (mean population doubling, 21.4) whilst another three goats received a membrane seeded with full-depth goat chondrocytes (mean population doubling, 3.5). After debridement of the margins of the lesion from the previous surgery the membrane was inserted into the defect and sutured with 8 individual suture points. The operated limb was immobilised for 2 weeks using a sling. The animals were sacrificed at 20 months after the second surgery. The operated knees were dissected, formalin fixed, decalcified and prepared for routine histology and immunocytochemistry as described above. Repair tissue grading was performed independently, in a blinded manner, by 3 researchers, based on the International Cartilage Repair Society (ICRS) scoring system.

## Results

### FACS analysis

**Figure 1:**
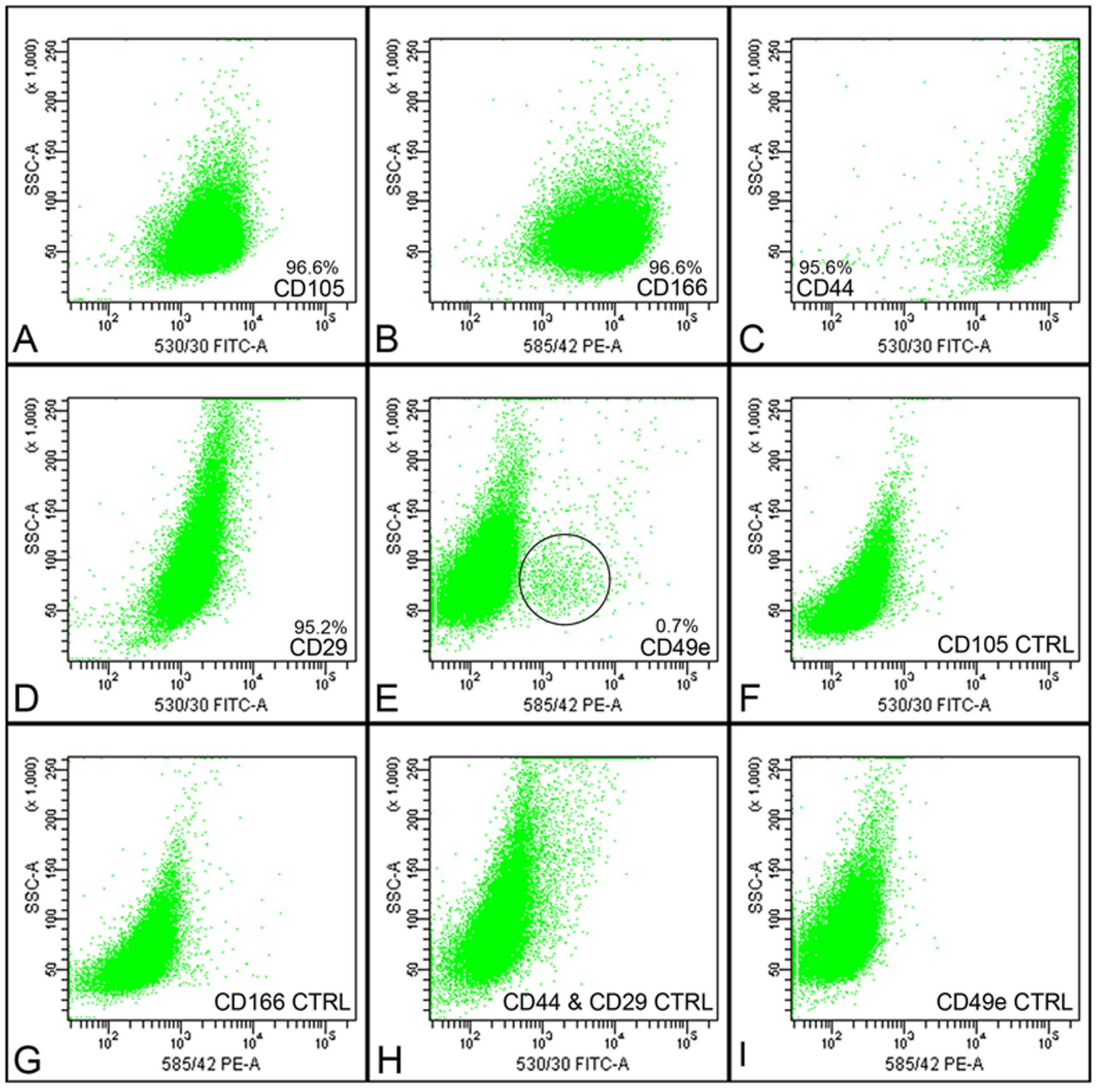
Flow cytometric analysis of full-depth chondrocytes. Full-depth chondrocytes were labelled for the putative stem cell surface markers CD105 (A), CD166 (B), CD44 (C), CD29 (D) and CD49e (E). Note the subpopulation of cells labelled for CD49e. Corresponding immunoglobulin control samples were analysed for each marker at each experimental run (F–I).

Fluorescently activated cell sorting (FACS) analysis was utilised to label the full-depth chondrocytes for a series of putative cartilage stem cell markers. Analysis of full-depth chondrocytes for CD105 (endoglin), CD166 (ALCAM), CD44 and CD29 (β1 integrin) all showed expression in over 95% of the viable cell population (Fig. 1A–1D). However, expression of CD49e, α5-integrin, was observed in a distinct population, 0.7%, within the viable full-depth chondrocyte population (Fig. 1E). On analysis of the clonal population, isolated using the fibronectin adhesion assay, almost 100% of the cells labelled for CD49e expression (data not shown).

### Fibronectin adhesion assay

**Figure 2:**
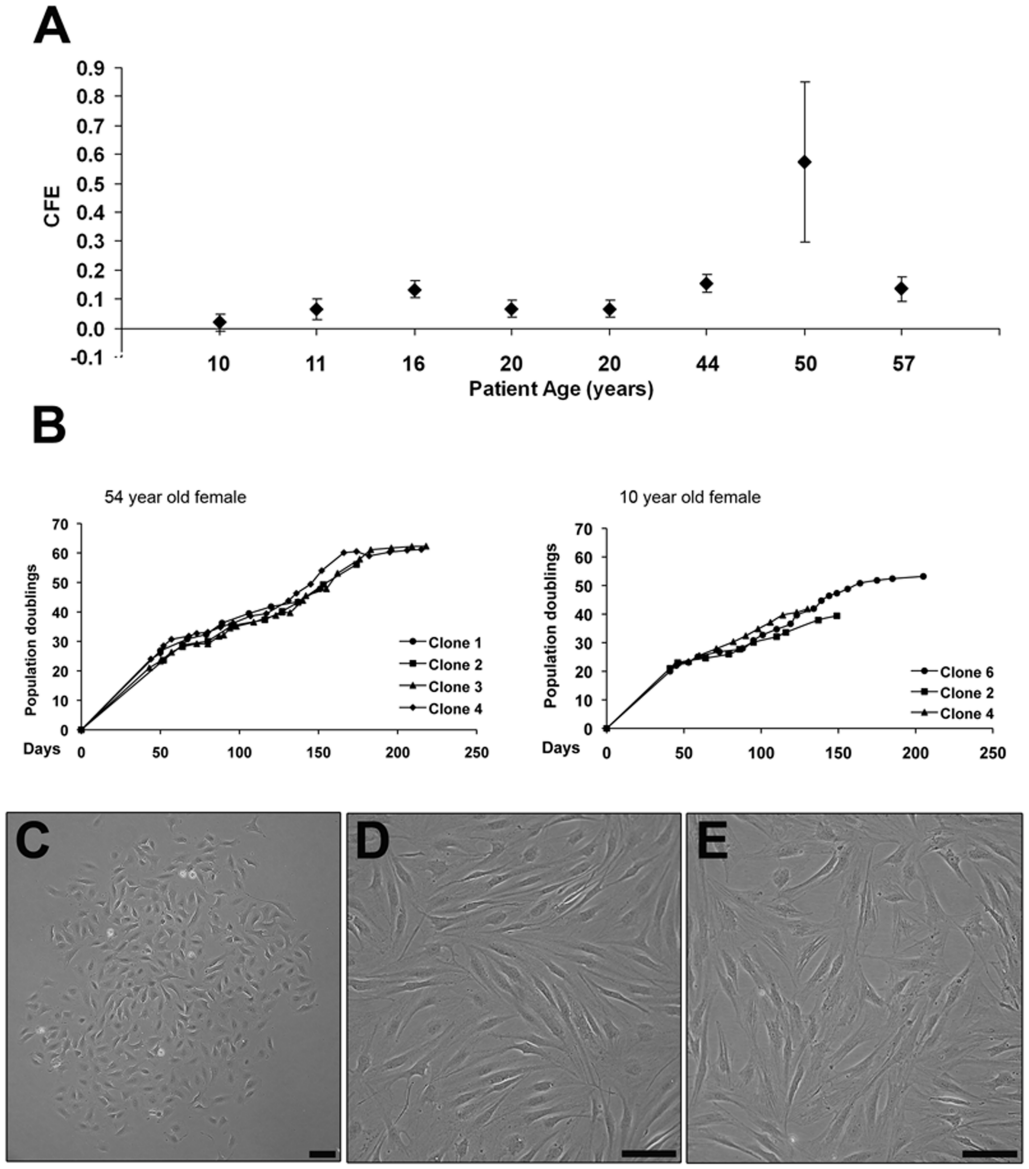
Population data from clonal cell lines. Colony forming efficiency calculated from 8 samples that were subjected to the fibronectin adhesion assay (as described in Materials & Methods) (A). Population doublings data from 2 representative samples demonstrating proliferative rate of the clonal cell lines during a period of over 200 days (B). Phase contrast microscopic appearance of a representative fibronectin adhered colony (C) and clonal monolayers at low (<30) and high (>30) population doublings (D & E respectively). Scale bar  =  100 µm.

Fifty percent of the samples showed a CFE (colony forming efficiency) of less than 0.1 with the remaining 40% demonstrating a CFE of 0.2 (Fig. 2A). Discrete colonies that form (comprising more than 32 cells) were selected and cultured in monolayer (Fig. 2C) and clonal cell proliferative capacity calculated. The cells were able to proliferate to over 60 population doublings, taking over 200 days (Fig. 2B). Clones obtained from the same sample proliferated at similar rates. Differences in growth kinetics were observed between the clones from different samples. Morphologically, the clonal cell lines showed a fibroblastic phenotype in early population doubling monolayer cultures that were retained in long-term cultures. There were no obvious differences in the size of the cells between early and late population doublings (Fig. 2D & E).

### Monolayer cultures

**Figure 3:**
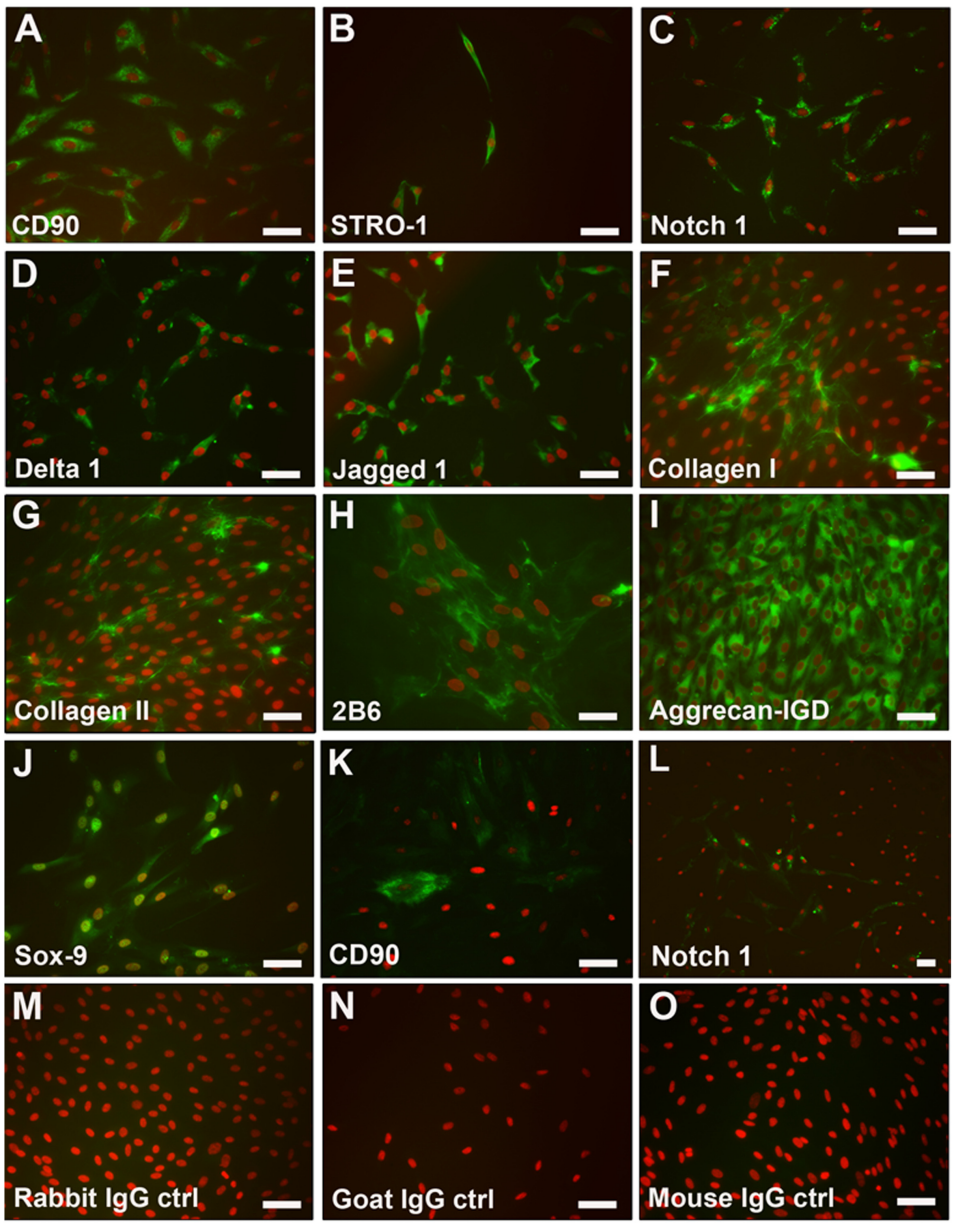
Phenotype of cartilage progenitor and full-depth chondrocyte cell lines. Monolayer cultures of expanded clonal cell lines at over 30 population doublings express the putative stem cell markers, CD90 (A) and STRO-1 (B) when localised using immunofluorescent labelling (green). Members of the Notch signalling family, Notch 1 (C), Delta 1 (D) and Jagged 1 (E) were expressed in clonal monolayer cultures. Markers of the chondrogenic phenotype, collagen type II (G), 2B6 (H), aggrecan-IGD (I), Sox9 (J), and also, collagen type I (F) were present in clonal monolayer cultures at over 30 population doublings. Full-depth chondrocytes show fewer cells with positive expression of CD90 (K) and Notch 1 (L) within the monolayer cultures. Corresponding immunoglobulin controls, rabbit (M), goat (N) and mouse (O), were all negative. Scale bars  =  50 µm.

Monolayer cultures of cartilage progenitors at population doublings greater than 30 demonstrated positive labelling for stem cell markers CD90 (Fig. 3A) and STRO-1 (Fig. 3B). Notch 1 receptor and its' corresponding ligands, Delta 1 and Jagged 1, which are involved in signalling pathways of stem cell differentiation, were also expressed in the cartilage progenitor monolayers (Fig. 3C–3E). Although expression of these putative stem cell markers were observed in full-depth chondrocytes, it was to a lesser extent (Fig. 3K, 3L). Analysis of chondrogenic phenotype markers, in monolayer clonal cell lines post-30 population doublings and subjected to chondrogenic induction by TGFβ2, resulted in the observed expression of collagen type I, collagen type II, chondroitin-4-sulphate (2B6), aggrecan-IGD (6B4+) and the chondrogenic transcription factor, Sox9 (Fig. 3F–3J). The induction of cartilage gene expression and immuno-detectable matrix synthesis in monolayer clonal cell lines after 30 population doublings is significant and noteworthy. Corresponding immunoglobulin controls for the antibodies were all negative (Fig. 3M–3O).

### Differentiation assays

**Figure 4:**
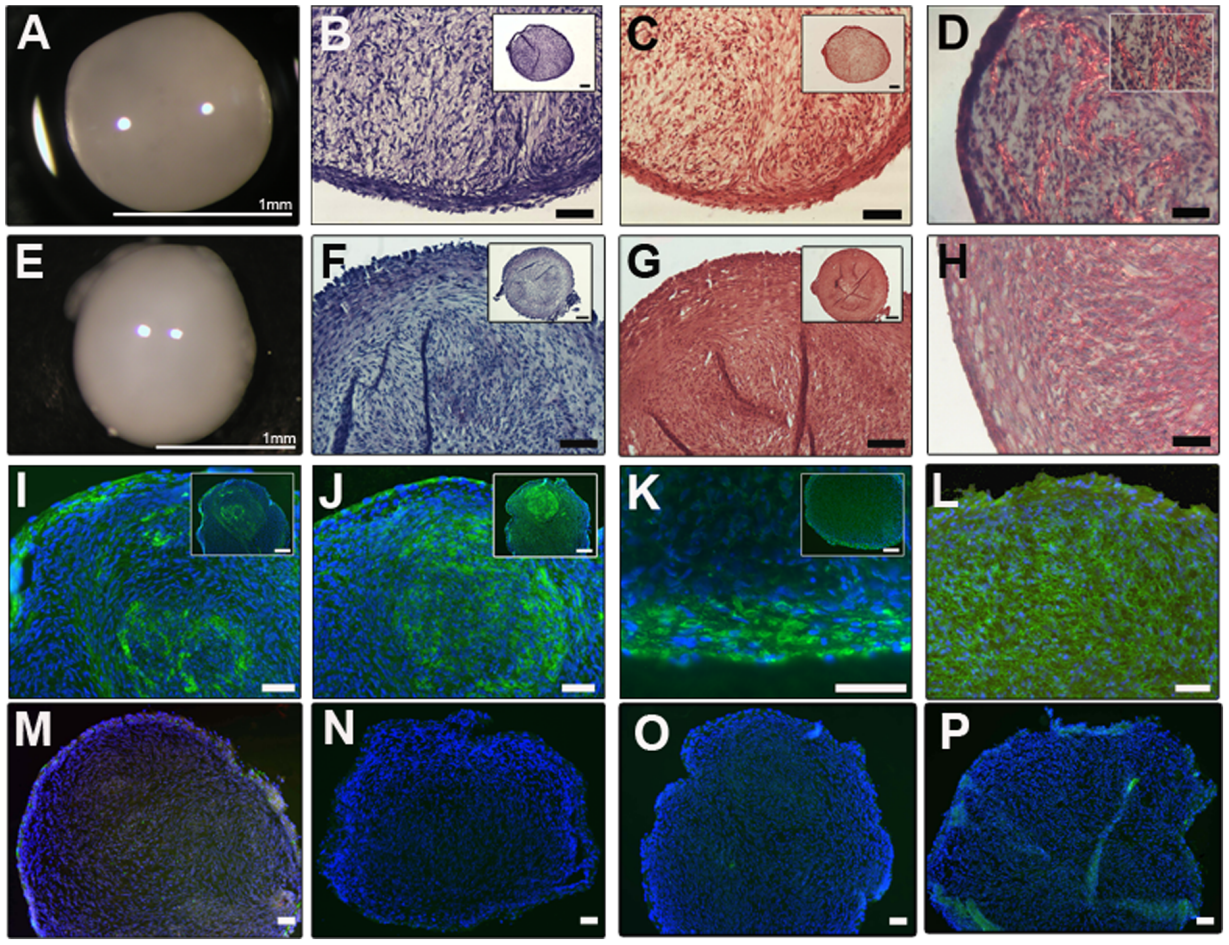
Chondrogenic differentiation of cartilage progenitor and full-depth chondrocyte populations. Gross morphology of a representative 3D cartilage progenitor (A) and full depth chondrocyte (B) pellet cultured in chondrogenic media for 21 days. Pellets display a shiny smooth surface. Toluidine blue (B, F) and safranin O (C, G) stained pellets demonstrate the presence of glycosminoglycans within the pellet matrix. Picro-sirius red staining highlights synthesis of collagen fibres in the pellet matrix (D, H). Immunohistochemistry of the clonal pellets demonstrates both collagen type I (I) and collagen type II (J) within the chondrogenic pellet matrix. Aggrecan labelling was present on the outer edge of the chondrogenic pellet (K) and 2B6 expression was present throughout the pellet matrix (L). Pellets cultured in chondrogenic media show no alkaline phosphatase (M) or collagen type X expression (N). Representative examples of negative controls for mouse monoclonal (O) and rabbit polyclonal (P) antibody protocols. Scale bars  =  50 µm.

At 30 population doublings or greater, clonal cells were pelleted into 3D Eppendorf cultures and maintained for 21 days in media containing factors to promote either chondrogenic or osteogenic differentiation. Four clonal cell lines and a full-depth chondrocyte sample cultured as pellets in chondrogenic differentiation media, both showed a smooth surface morphology (Fig. 4A, 4C). Toluidine blue and safranin O staining demonstrated glycosaminoglycan synthesis 21 days after chondrogenic culture (Fig. 4B, 4C, 4F, 4G). The presence of collagen fibrils within the cartilage progenitor pellet matrix was indicated by picro-sirius red staining (Fig. 4D) and on further analysis, detection of collagen type I (Fig. 4I) and collagen type II (Fig. 4J), by immunohistochemical methods, was revealed and indicated a differentiation process similar to that found during early development [44]. Positive labelling for aggrecan core protein, 6B4, was observed intensely in the outer regions of the pellet with weaker labelling demonstrated in central regions (Fig. 4K). Labelling for 2B6 was present throughout the pellet matrix (Fig. 4L). Significantly, neither alkaline phosphatase (Fig. 4M) nor collagen type X (Fig. 4O) were observed in pellets cultured in chondrogenic media.

**Figure 5:**
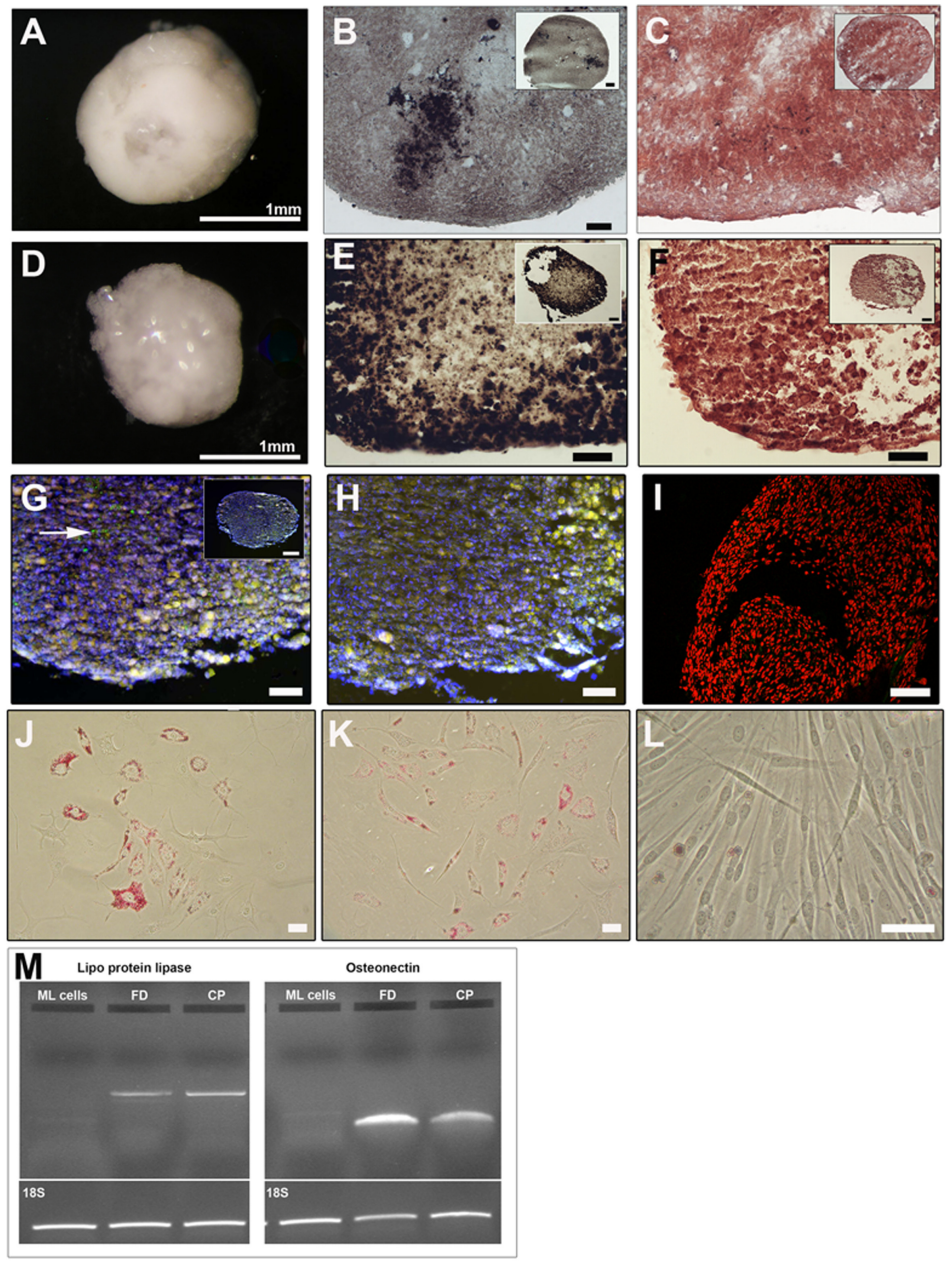
Lineage differentiation of clonal and full depth cell populations. Pellets cultured in osteogenic differentiation media show a rough surface topography in the full depth chondrocytes (A) and in cartilage progenitor cell pellets (D). Regions of mineralisation are indicated in the pellets cultured in osteogenic media by von Kossa (B, E) and alizarin red staining (C, F). Alkaline phosphatase (G;arrowed) was present in a small region of the osteogenic cartilage progenitor cultured pellets and collagen type X was absent (I). Representative example of a negative control for mouse monoclonal antibody protocol (H). Monolayer clonal cells cultured in adipogenic differentiation media show positive staining with Oil Red O in both cartilage progenitor cells (J) and full depth chondrocytes (K). Control cultures did not stain with Oil Red O (L). Scale bars  =  50 µm. mRNA expression for lipo-protein lipase (bp 505) and osteonectin (bp 107) in non-treated monolayer (ML) chondrocytes, treated full-depth chondrocytes (FD) and treated cartilage progenitors (CP) (M).

The cartilage progenitor pellets cultured in osteogenic differentiation media showed a rough surface topography and were slightly smaller in size compared to the full-depth chondrocyte pellets (Fig. 5A, 5D). When stained for mineralisation, cartilage progenitor pellets showed extensive regions of calcium deposition as indicated by both von Kossa (Fig. 5E) and alizarin red stain (Fig. 5F). The full-depth chondrocyte pellets displayed only small regions of mineralisation (Fig. 5B). PCR analysis for osteonectin, a marker for early bone development, demonstrated mRNA expression in both the full-depth and cartilage progenitor pellets (Fig. 5M). Osteogenic differentiation was not evident as alkaline phosphatase activity (Fig. 5G) was seen at a very low level and collagen type X (Fig. 5I) expression was entirely absent in the cartilage progenitor pellets.

Clonal cells and full-depth chondrocytes cultured as monolayers in adipogenic media stained positively for lipid deposition (Fig. 5J, 5K) whereas those cultured in control media did not (Fig. 5L). PCR analysis for lipo-protein lipase showed mRNA expression in the full-depth and cartilage progenitor population (Fig. 5M).

### Cytogenetic Analysis

**Figure 6:**
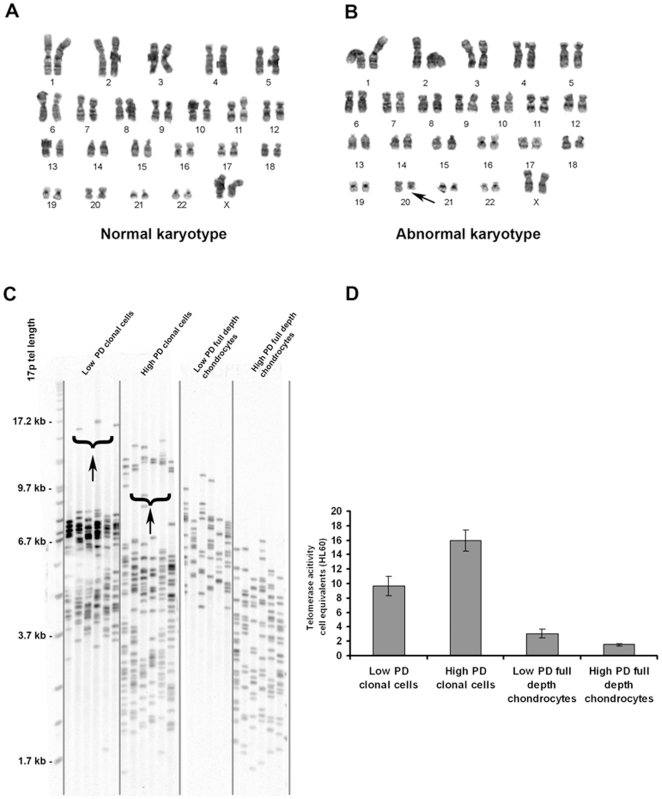
Cytogenetic Analysis. Normal female, 46, XX karyotype was observed in a clonal cell line at 31.3 population doublings (A). In one flask from the same cell line, 2 out of 12 cells displayed an abnormal karyotype; a deletion on the long arm of chromosome 20 at q11.2 (B; arrowed). **Telomere length analysis and real-time quantitative telomere repeat amplification procedure (RTQ-TRAP) of telomerase activity in clonal cell lines and full-depth chondrocyte populations.** STELA reveals both clonal cell lines and full-depth chondrocytes undergo telomere erosion (C). It is interesting to note a subpopulation of cells in the clonal cell line show a larger distribution which is increasing with time in culture (arrowed). RTQ-TRAP analyses of telomerase activity in HL60 cell equivalents showed low population doubling (<30) clonal cells displaying a 3.1-fold greater activity than low population full depth chondrocytes (D). High population doubling clonal cells show a 10.6-fold greater activity than full depth chondrocytes at a high population doubling (>30). PD  =  population doubling.

Examination of the GTG-banded preparations from 3 flasks from a clonal cell line from one specimen (54 year old, PD = 31.3) revealed a normal female karyotype, 46, XX (Fig. 5A). However, 2 of 12 cells examined from one of the culture flasks showed a terminal deletion of the long arm of chromosome 20, from q11.2 to qter (Fig. 6B). This anomaly was not observed in the other 2 culture flasks. The clonal cell line from the second specimen (10 year old, PD = 23.5) showed a normal female 46, XX karyotype in 70 cells examined. However, 10 of the 70 cells examined had an apparently balanced translocation involving the short arm of chromosome 7 and the long arm of chromosome 16. The breakpoints appeared to be at p13 and q22 respectively. The karyotype from the second clonal cell line (PD = 28.5) from the second specimen showed a normal female karyotype, 46, XX in 50 cells examined from 2 flasks.

### Telomere length and telomerase activity analysis

Two clonal cell lines were analysed, one of which is depicted for greater clarity although data from both were very similar. The clonal cell line and the full-depth chondrocyte population undergo telomere erosion with progressive cell divisions (Fig. 6C). It is interesting to note that the distributions within the clonal cell lines display less heterogeneity that is normally observed in bulk populations of cells or tissues where a typical SD of around 3 kb would be expected. In the clonal cell line, it is apparent that subsets of cells with a larger distribution are present and the proportion of cells within this subset is increasing with time in culture (Fig. 6C, arrowed). Full-depth chondrocytes displayed almost baseline levels of telomerase activity in both low (PD12) and high (PD49) population doubling cultures (Fig. 6D). The clonal cell line at a high population doubling (PD39) exhibited the greatest telomerase activity, even higher than that of the low population doubling (PD26) clonal cell line, possibly due to the presence of a subset of cells showing a high telomere length. When converted to HL60 cell equivalent units, the clonal cell line showed 3.1-fold greater telomerase activity than full-depth chondrocytes at low population doubling and at the high population doubling this figure increased to a 10.3-fold difference.

### Engraftment of cartilage progenitor cell lines

**Figure 7:**
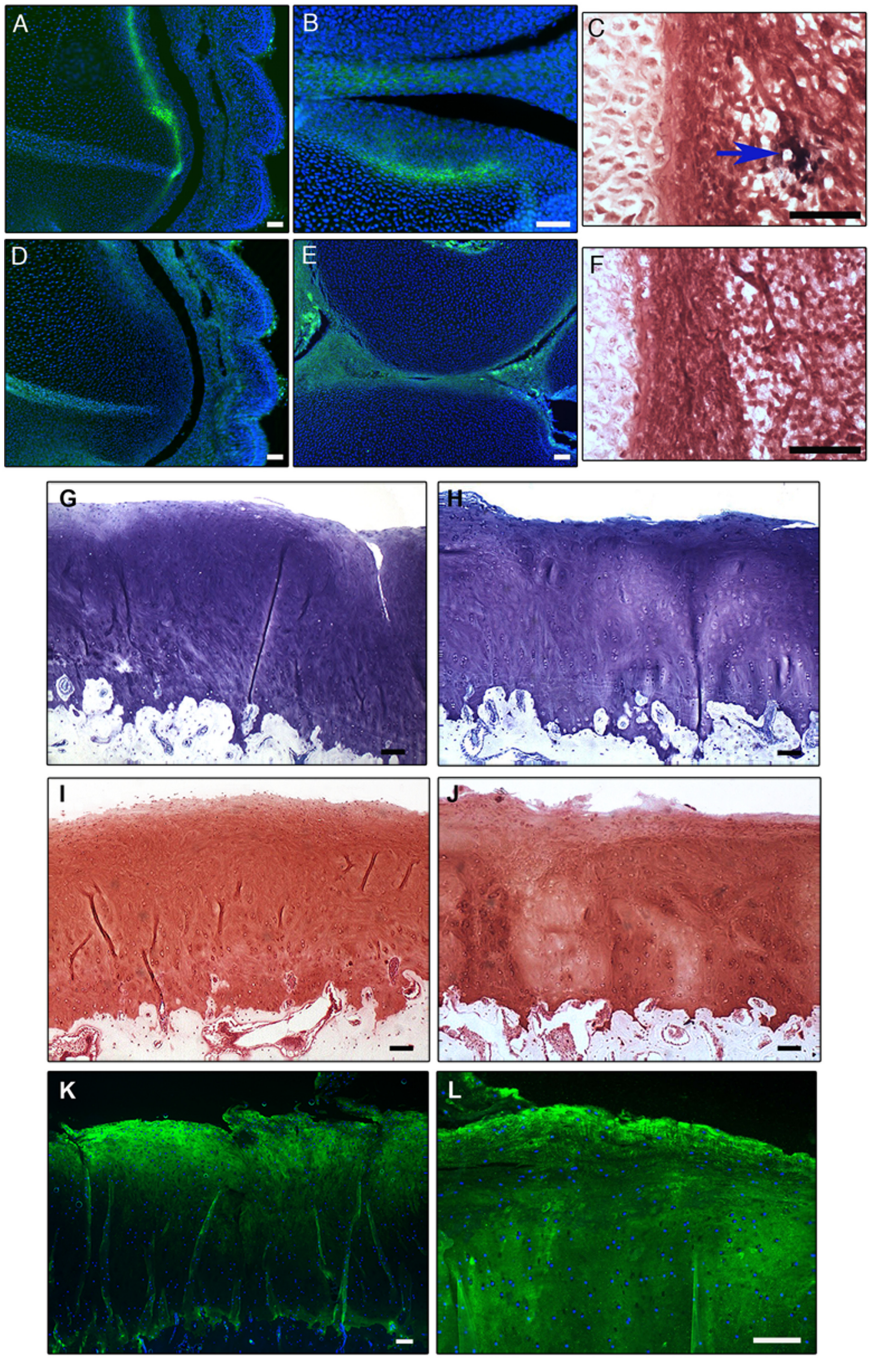
Engraftment of human cartilage progenitor cells into developing chick hind limbs. Cells expressing human collagen type I are present in the growth plate of the developing chick limb at st36 (A). The high power image demonstrates human collagen type I expression in the surface region of the developing cartilage anlagen (B). IgG negative control (D). Expression of chick collagen type I is not evident in st36 chick limb (E). ISH for human Alu repeats on a representative section from a st36 chick limb (C). The Alu-positive nuclei are stained black (arrowed). Negative control for ISH for the Alu repeats (F). Scale bars  =  50 µm. ***In vivo*** **implantation of goat chondroprogenitors into cartilage defects.** Histological stained sections of repair tissue in the caprine *in vivo* repair model. Toluidine blue stained sections of repair tissue containing the membrane seeded with full-depth chondrocytes (G) and chondroprogenitors (H) shows examples of an integrated repair tissue. Safranin O staining demonstrated proteoglycan synthesis in the repair tissue in defects treated with full-depth chondrocyte seeded membranes (I) and chondroprogenitor seeded membranes (J). Repair tissue in defects treated with chondrocyte seeded membranes (K) or chondroprogenitor seeded membranes (L), labelled positive for collagen type II. Scale bars  =  100 µm.

In order to test for *in vivo* plasticity, cartilage progenitor cells were injected into st23 chick hind limbs. A high number of cells labelling positively for human collagen type I were observed in the perichondrium and alongside the prehypertrophic region of the developing anlagen (Fig. 7A, 7B). Controls determined that the human collagen type I antibody was specific for human collagen type I and did not recognise chick collagen type I or other chick collagens (Fig. 7D, 7E). *In situ* hybridisation for human Alu repeats, on a representative section from a chick limb 7 days after human clonal cell transplantation showed human Alu-positive nuclei stained black in the peri-articular tissues (Fig. 7C). Control sections were negative (Fig. 7F).

### Caprine *in vivo* repair study

In order to determine whether articular cartilage progenitors are suitable candidates for cartilage repair, we carried out a proof of principle pilot study on goats. Chondroprogenitors obtained from the goat study demonstrated an average CFE of 0.2. Positive collagen type II and aggrecan labelling was observed by both immunohistological and PCR analysis in the 3D chondrogenic pellet cultures.

The ICRS scoring system designed by Brittberg and Peterson [45] was used to evaluate cartilage repair in the goat tissue sections. Histological repair scores for full-depth chondrocytes ranged from 5 (abnormal) to 10 (nearly normal) whilst repair scores for chondroprogenitors ranged from 7 (abnormal) to 10 (nearly normal). Overall, full-depth chondrocytes had a mean score of 7.8 and chondroprogenitors had a mean score of 8.2 thus suggesting no overall differences between the two experimental groups. Examples of excellent lateral integration in both full-depth chondrocyte (Fig. 7G, 7I) and chondroprogenitor (Fig. 7H, 7J) treatment groups were observed. In some of the samples, there was no obvious demarcating border and it was difficult to tell where the defect actually was. In all of the samples, both full-depth chondrocyte (Fig. 7K) and chondroprogenitor seeded membranes (Fig. 7L) showed evidence of collagen type II positive repair tissue.

## Discussion

Previous research has relied on cell surface markers, originally designated to characterise bone marrow stromal cells, as a means of isolating possible stem cell populations from articular cartilage [27], [29], [46]. In particular, research suggested that CD105 and CD166 as possible candidates of cartilage stem cell markers but our study and others have demonstrated that a sub-population of cartilage stem cells cannot be isolated from these markers alone as they are widely expressed by mature chondrocytes in the native tissue [29], [30], [47]. Isolating a possible stem cell population reliant on stem cell marker expression, however, could prove problematic for cell-based repair therapies. In a clinical situation, isolation of the correct cell population in suitable numbers would be dependent on an extremely consistent method of cartilage digestion and culture conditions, as these parameters play a significant role in cell surface marker expression. Indeed, work from our own laboratory and others have demonstrated that these putative markers vary during culture [48]. Additionally, clinically accredited FACs machines have yet to come on-stream.

In light of these studies, we decided to isolate what we now characterise as a cartilage progenitor population using the fibronectin adhesion assay, selecting for discrete colonies (of over 32 cells to eliminate transit amplifiers) which could then be expanded to high numbers [33]. Currently, generation of high numbers of chondrogenically competent full-depth chondrocytes for cell-based repair strategies is not achievable due to the loss of Sox-9 expression and concomitant loss of the cartilage phenotype [26], [49]. Expansion of our cartilage progenitor clonal populations to a high population doubling suggests that this method of isolation is indeed selecting for cartilage progenitor populations.

It is interesting to observe that a sub-population (0.7%) of cells within the total chondrocyte cell mass express CD49e (α5 integrin) and indeed the clonal cell lines obtained by adhesion to fibronectin express this marker (99%) when expanded. The expression of this marker may explain the high affinity of these progenitor cells for fibronectin. Whether or not CD49e can be utilised as a specific marker for this cartilage progenitor cell population remains to be determined. However, the colony forming efficiency of these cells indicates that they are present at a ratio of 1:100, which is in the order of 0.7%.

The cytogenetic analyses demonstrated that cartilage progenitors that had undergone 31.3 population doublings are largely normal but with some anomalies that are likely due to the high PD number and culture conditions. This was particularly apparent in the deletion of the short arm of chromosome 20 in one flask yet not apparent in the other two flasks containing the same clonal cell line. It can be considered cautionary and underlies the necessity of carrying out a karyotype analysis when generating cells for clinical application.

It is well documented that when full depth chondrocytes in monolayer culture lose their rounded cell configuration, there is a loss of phenotype associated with the number of division cycles the cell undergoes and the eventual loss of Sox9 expression [5], [50], [51], [52], [53]. Monolayer cartilage progenitor populations that have been isolated in this study, although demonstrating a flattened morphology in culture, show expression of putative stem cell markers, yet when induced to undergo chondrogenic differentiation by addition of TGFβ2, markers of the chondrogenic phenotype are expressed and translated into immuno-detectable proteins. Using the bovine equivalent progenitor cells, we have demonstrated that the extended chondrogenic potential of these cells is related to the maintenance of Sox9 expression during extended monolayer culture and we would suggest that this is likely with the human equivalent cells [26].

Another effect of long-term chondrocyte culture is cell senescence due to a shortening of telomeres, the DNA sequences necessary for chromosome replication [54], [55], [56]. As true stem cells maintain their telomere length and show unlimited replicative capacity, this provided a way of characterising the isolated cartilage progenitor population and also strengthened the case for their suitability for cell-based repair therapies [57]. Full-depth chondrocytes and cartilage progenitors investigated in our study displayed eroded telomeres with time in culture consistent with growth kinetic data. However, if we study the results in more detail, a sub-population of cells within the cartilage progenitor population is observed that maintain their telomere length. Indeed, this sub-population of cells maintains their telomere length and increases in number with time in culture. However, we do not find this sub-population expanding beyond 60 PDs and may senesce in a telomere independent fashion.

The cartilage progenitor population express a number of chondrogenic markers and matrix components when induced to undergo chondrogenesis *in vitro*, including collagen type I and collagen type II. In culture, collagen type I is often considered an indicator of fibrocartilage. However, the expression of collagen type II in the chondrogenic cell pellets suggests that the cartilage progenitors are following a developmental process in their matrix synthesis, synthesising firstly collagen type I and secondly collagen type II as the cells mature [44]. Evidence of co-localisation of the two collagens at the articular surfaces was described in a study by Archer *et al* [58] and it is interesting to note that collagen type 1 is observed in the pellets in the outer layer of cells, which appear flattened – similar to the flattened interzone cells and perichondrium in the developing limb, as described in the same study. Osteogenically induced cultures show signs of mineralisation when cultured as a 3D pellet. However, there is no evidence of bone formation *per se*; neither collagen type X protein expression nor alkaline phosphatase activity were evident in the chondrogenic or osteogenically induced 3D cultured pellets. These data highlight that cartilage progenitors neither undergo chondrogenic terminal differentiation nor true osteogenesis. Notably, many published studies assay osteogenesis of stem cells in monolayer culture through analysis of expression of alkaline phosphatase and the appearance of calcification by von Kossa staining that *de facto*, is not an assay for bone formation. Indeed, if the cells are demonstrating a restricted differentiation potential *in vitro*, then the ability of these cells to form stable ectopic cartilage that doesn't undergo terminal differentiation *in vivo* would be a very desirable factor for using these cells in clinical applications for cartilage repair strategies. In contrast, current research into BMSCs for cartilage regeneration procedures demonstrates terminal differentiation and extracellular matrix (ECM) calcification *in vitro* upon chondrogenic induction, ultimately resulting in failure of transplantation [16], [22], [59], [60].

Multi-lineage potential is often used to characterise a mesenchymal stem cell population. The results from our study demonstrate that the cartilage progenitor cells have the capacity to differentiate into the adipogenic lineage, whilst retaining a restricted differentiation potential during osteogenic differentiation, as discussed above. However, the full-depth chondrocytes are also positive for differentiation into these lineages, albeit to a lesser extent. This can be explained as such; during the isolation procedure, progenitor cells are part of the full-depth chondrocyte culture and are likely to have become enriched during the cell expansion process. This small progenitor population within the full-depth chondrocytes would, therefore, undergo differentiation when cultured in the appropriate media, as we have observed.

Characteristically, mesenchymal stem cells can migrate and engraft into tissues, including regions of injury, and undergo site-specific differentiation [61], [62], [63]. It is encouraging to see that data from our engraftment studies demonstrated that the cartilage progenitor population can migrate to its specific milieu within the developing limb. The isolated cartilage progenitor populations largely engrafted within the perichondrium – an already known niche of stem cells and the cells also migrated to the surface of the articular cartilage – where data by Dowthwaite *et al* [25] has demonstrated the presence of a progenitor population. However, engrafted cells were observed in other connective tissues including dermis but were not seen outside tissues of the connective tissue lineage, lending further support to progenitor status.

To determine the potential of chondroprogenitors in *in vivo* cartilage repair, we have used the goat as our model. The goat chondroprogenitors were isolated and characterised in the same manner as the human chondroprogenitors. The caprine *in vivo* repair study revealed examples of excellent integration in cartilage defects treated with Chondro-Gide® membrane seeded with goat chondroprogenitors. When subjected to the ICRS scoring system to evaluate cartilage repair, however, there was no difference in repair between defects that were treated with chondroprogenitors and those treated with the current gold standard of expanded full-depth chondrocytes. Although, no differences in repair tissue were observed at the 20-month end point of the study, the main advantage of these cells over full-depth chondrocytes is the ability to generate very large cell numbers that retain their phenotype, from a single cell. As a result, it would be feasible to treat much larger defects than is currently possible.

### Conclusion

The main findings from these data lead to the conclusion that human articular cartilage contains a cartilage progenitor population that retains a stem cell-like phenotype until appropriate induction of chondrogenic differentiation is required. As such, we propose that this cell population is an ideal candidate to use in the development of enhanced protocols for future cell-based tissue engineering procedures that require cells with an extensive replication capacity and restricted differentiation potential. As a single cell can generate millions of cells that retain their chondrogenic phenotype *in vivo*, this isolation procedure would ensure sufficient numbers of cells, that maintain their ability to form cartilage, are readily available for surgical procedures. These characteristics are favourable if the transplanted cells are to physically contribute to the repair and maintenance of the cartilage defect long-term.

## Supporting Information

Text S1 Immunohistochemistry.
(0.04 MB DOC)

Text S2 Cytogenetic Analysis.
(0.04 MB DOC)

Text S3 Telomere length analysis.
(0.04 MB DOC)

Text S4 RTQ-TRAP.
(0.04 MB DOC)
