# Supplementary material for: Identification and Clonal Characterisation of a Progenitor Cell Sub-Population in Normal Human Articular Cartilage
Source: PLoS One. 2010 Oct 14;5(10):e13246. doi: 10.1371/journal.pone.0013246 (PMC2954799; doi:10.1371/journal.pone.0013246)
Supplement: Text S1 — Immunohistochemistry. (0.04 MB DOC) [file pone.0013246.s001.doc]

**Text S1**

**Immunohistochemistry**

Monolayer cultures were fixed in cold 95% ethanol or 4% paraformaldehyde for 10 minutes and washed in PBS. Cells were incubated with 0.1% Triton-X for 5 mins. Embedded pellet sections and monolayer cells were blocked with the appropriate species-specific serum for 30 minutes at room temperature then excess serum removed and cells were incubated with the primary antibodies at a concentration of 10µg ml^-1^ in PBS overnight at 4°C. Cells were washed in PBS and incubated with appropriate FITC‑conjugated secondary antibodies (1µg ml^-1^) for 1 hour at room temperature then mounted in Vectashield containing PI (Vectorlabs, UK). At each experimental run, the appropriate immunoglobulin (10µg ml^-1^) replaced the primary antibody, as a negative control. For 6B4+, 2B6, collagen type II and collagen type X, pellet sections and monolayer cells were subjected to a chondroitinase (0.25U ml^-1^; Sigma, UK) and hyaluronidase (2U ml^-1^; Sigma, UK) pre-treatment for 1 hour at 37ºC. For collagen type I, pellet sections and monolayer cells were subjected to Proteinase K (2.0µg ml^-1^) digest for 15 mins.
