# Supplementary material for: Identification and Clonal Characterisation of a Progenitor Cell Sub-Population in Normal Human Articular Cartilage
Source: PLoS One. 2010 Oct 14;5(10):e13246. doi: 10.1371/journal.pone.0013246 (PMC2954799; doi:10.1371/journal.pone.0013246)
Supplement: Text S2 — Cytogenetic Analysis. (0.04 MB DOC) [file pone.0013246.s002.doc]

**Text S2**

**Cytogenetic Analysis**

When clonal cells were almost confluent cultures were exposed to 5’-Bromo-2’-deoxyuridine (BrdU; Sigma, UK) and Colcemid (Invitrogen, UK) overnight. Culture medium was removed and replaced with Amniochrome II medium (BioWhittaker, UK Ltd) containing BrdU at a concentration of 37.5µg ml^-1^ and Colcemid at a concentration of 0.6µg ml^-1^. Cultures were gassed with 90% N_2_ / 5% O_2_ / 5% CO_2_ (BOC) and incubated at 37ºC overnight. After exposure to Colcemid / BrdU, medium was removed and cultures were washed with PBS. PBS was poured off and 1ml of trypsin / EDTA (Invitrogen, UK) pre-warmed to 37ºC was added to detach and dissociate cells in the flasks. After 3 mins, 2.8ml of hypotonic solution [1ml FBS : 9ml sterile water] was added for 10 mins at room temperature. The cell suspensions were then fixed using 3:1 methanol-acetic acid, AnalaR, (BDH, UK) fixative. Five drops of methanol-acetic acid fixative were added followed by a further 2ml added slowly whilst gently mixing. Cell suspensions were then transferred into centrifuge tubes and centrifuged at 1500rpm for 10 mins. Centrifugation and change of fixative (approximately 5ml) was repeated three times, using 2:1 methanol-acetic acid fixative for the last fixation.

Following centrifugation the pellet of fixed material was re-suspended in 0.5ml-1.5ml 2:1 methanol-acetic acid fixative and spread on slides. A drop of the suspension was run onto a wet microscope slide and allowed to evaporate at a relative humidity of approximately 50%.

Giemsa banding (GTG-banding) was accomplished using a modified method. Slides, aged for 3-5 days at room temperature, were incubated for 8-10 seconds in trypsin solution, rinsed in 3 changes of normal saline, (sodium chloride 0.9%) then stained in 10% Giemsa stain (R A Lamb, UK) in phosphate buffer pH6.8 (BDH, UK) for 1.5 mins. Slides were then rinsed in 3 changes phosphate buffer pH6.8, dried and mounted in Entellan mountant (Merck, Nottingham, UK).
