# Supplementary material for: Identification and Clonal Characterisation of a Progenitor Cell Sub-Population in Normal Human Articular Cartilage
Source: PLoS One. 2010 Oct 14;5(10):e13246. doi: 10.1371/journal.pone.0013246 (PMC2954799; doi:10.1371/journal.pone.0013246)
Supplement: Text S3 — Telomere length analysis. (0.04 MB DOC) [file pone.0013246.s003.doc]

**Text S3**

**Telomere length analysis**

From the full-depth chondrocytes and clonal cells, DNA was extracted using standard proteinase K, RNase A, phenol / chloroform protocols, solubilized the DNA by digestion with *EcoRI*, and quantified in triplicate by Hoechst 33258 fluorometry (BioRad Laboratories, Hertfordshire, UK). The genomic DNA was diluted to 10ng µl^-1^ in 10mM Tris-HCl pH 7.5. Ten ng of DNA was further diluted to 250 pg µl^-1^ in a volume of 40µl containing 1µM Telorette2 linker and 1 mM Tris-HCl pH 7.5. Multiple PCRs (typically 6 reactions per sample) were carried out for each test DNA in 10µl volumes containing 250pg of diluted DNA, 0.5µM of the telomere-adjacent and Teltail primers, 75mM Tris-HCl pH8.8, 20mM (NH_4_)_2_SO_4_, 0.01% Tween-20, 1.5mM MgCl_2_, and 0.5 U of a 10:1 mixture of Taq (ABGene, UK) and Pwo polymerase (Roche, UK). The reactions were cycled with an MJ PTC-225 thermocycler (MJ research, USA) as described previously. The DNA fragments were resolved by 0.5% TAE agarose gel electrophoresis, and detected by two separate Southern hybridizations with a random-primed α-^33^P labelled (GE Healthcare, UK) telomere repeat containing probe and telomere-adjacent probe together with a probe to detect the 1kb (Stratagene, UK) and 2.5 kb (BioRad Laboratories, UK) molecular weight markers. The hybridized fragments were detected by phosphor-imaging with a Molecular Dynamics Storm 860 phosphorimager (GE Healthcare, UK). The molecular weights of the DNA fragments were calculated using the Phoretix 1D quantifier (Nonlinear Dynamics, UK).
