# Supplementary material for: Identification and Clonal Characterisation of a Progenitor Cell Sub-Population in Normal Human Articular Cartilage
Source: PLoS One. 2010 Oct 14;5(10):e13246. doi: 10.1371/journal.pone.0013246 (PMC2954799; doi:10.1371/journal.pone.0013246)
Supplement: Text S4 — RTQ-TRAP. (0.04 MB DOC) [file pone.0013246.s004.doc]

**Text S4**

**Real Time Quantitative Repeat Amplification Procedure (RTQ-TRAP)**

Cells were lysed at a concentration of 1x10^6^ per 200µl of lysis buffer; 10 mM Tris pH 8.3, 1.5 mM MgCl_2_, 1 mM EGTA, 10% glycerol, 0.5% CHAPS buffer, 1 mM PMSF, 5mM ß-mercaptoethanol and 200U RNAsin. Each reaction comprised 1 x SYBR Green Mastermix (Primer Design, UK), 10 mM EGTA, 0.2µg T4 gene protein, 0.3µM primers TS (5’-AAT CCG TCG AGC AGA GTT-3’) and ACX (5’-GCG CGG [CTT ACC]_3_ CTA ACC-3’) and 25 x 10^3^ cells in a final volume of 25µl. The reaction mixture was incubated for 25°C for 20 mins to allow the telomerase to extend the TS primer, heated to 95°C for 10 mins to activate Taq Polymerase, followed by 40 cycles at 95°C for 20s, 50°C for 30s and 72°C for 90s. Telomerase activity in cell samples was calculated based on the threshold cycle (C_T_). All samples were run in triplicate and lysis buffer was used as a negative control. Immortal human leukaemia cell line HL60 was used as a cell-line positive for telomerase activity. HL60 cell extracts were serially diluted and assayed by RT-TRAP to generate a standard curve that was used to derive telomerase activity units based on the number of input cell equivalents.
